# Effects of Magnesium, Calcium, and Aluminum Chelation on Fluoroquinolone Absorption Rate and Bioavailability: A Computational Study

**DOI:** 10.3390/pharmaceutics13050594

**Published:** 2021-04-21

**Authors:** Daniel M. Walden, Maksim Khotimchenko, Hypatia Hou, Kaushik Chakravarty, Jyotika Varshney

**Affiliations:** VeriSIM Life, San Francisco, CA 94104, USA; daniel.walden@verisimlife.com (D.M.W.); maksim.khot@verisimlife.com (M.K.); hypatia.hou@verisimlife.com (H.H.); kaushik.chakravarty@verisimlife.com (K.C.)

**Keywords:** fluoroquinolones, antibiotics, antacids, multivalent metals, pharmacokinetics, bioavailability, oral absorption, molecular modeling

## Abstract

Fluoroquinolones (FQs) are a widespread class of broad-spectrum antibiotics prescribed as a first line of defense, and, in some cases, as the only treatment against bacterial infection. However, when administered orally, reduced absorption and bioavailability can occur due to chelation in the gastrointestinal tract (GIT) with multivalent metal cations acquired from diet, coadministered compounds (sucralfate, didanosine), or drug formulation. Predicting the extent to which this interaction reduces in vivo antibiotic absorption and systemic exposure remains desirable yet challenging. In this study, we focus on quinolone interactions with magnesium, calcium and aluminum as found in dietary supplements, antacids (Maalox) orally administered therapies (sucralfate, didanosine). The effect of FQ–metal complexation on absorption rate was investigated through a combined molecular and pharmacokinetic (PK) modeling study. Quantum mechanical calculations elucidated FQ–metal binding energies, which were leveraged to predict the magnitude of reduced bioavailability via a quantitative structure–property relationship (QSPR). This work will help inform clinical FQ formulation design, alert to possible dietary effects, and shed light on drug–drug interactions resulting from coadministration at an earlier stage in the drug development pipeline.

## 1. Introduction

**Figure 1:**
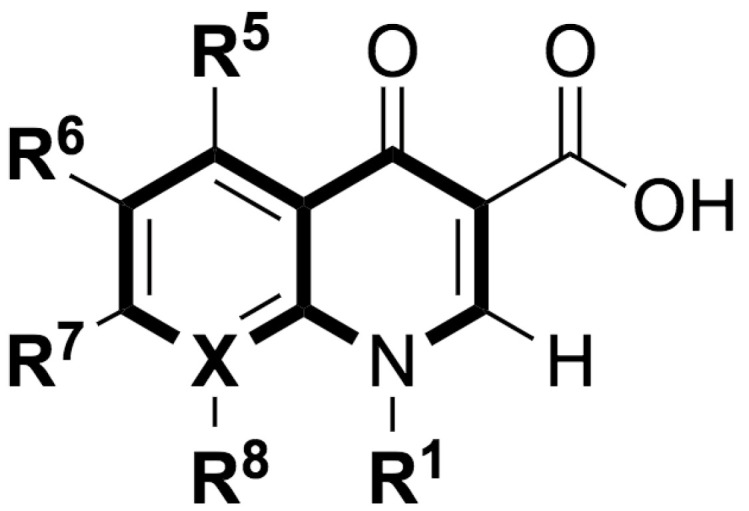
Shared core scaffold of quinolone antibacterial compounds. X = N indicates 1,8-naphthyridine, X = C defines quinolones. R^6^ = F defines fluoroquinolones.

Quinolones comprise a widely prescribed class of antibiotics against both Gram-negative and Gram-positive bacterial infections. In 2018, ciprofloxacin alone was prescribed over 6.7 million times in the United States, representing the 109th most prescribed medication in the country [1]. Significant attention to quinolone development followed the discovery of the antibacterial properties of nalidixic acid, itself a by-product of chloroquine synthesis. As the spearheading member of the first-generation quinolones, nalidixic acid was approved to treat Gram-negative (primarily *Escherichia coli*) urinary tract infections (UTIs) and began clinical applications in 1967, yet ultimately saw limited use due to its narrow spectrum and high dosage requirements for efficacy [2]. Over the last five decades, quinolone development and structure–activity relationships have focused on expanding the antimicrobial spectrum and overall activity while also improving compound pharmacokinetics (PK) and reducing both toxicity and drug interactions [3,4]. Nalidixic acid, while technically a 1,8-naphthyridine derivative, exhibits the defining core 4-quinolone bicyclic ring system shared by quinolone therapeutic compounds (Figure 1). Critical substituents for high potency are hydrogen substitution at position-2, a carboxylic acid at position-3, and a ketone at position-4 [5,6]. Fluorine substitution at C6 was identified as key to increased antibacterial potency and tissue penetration [7]. The C6 fluorine substitution to the initial quinolone scaffold is now recognized as a critical pharmacophoric component, and this subclass, termed fluoroquinolones (FQs), comprise most contemporary quinolones in development and with clinical applications.

Reduction in oral bioavailability due to chelation between FQs and multivalent metals is a well-established phenomenon with numerous studies disclosing disruptive interactions between quinolones and metal cations [8]. Nix et al. studied the effect of aluminum and magnesium from Maalox antacid on ciprofloxacin PK. The maximum plasma concentration (*C*_max_) and area under the plasma concentration-versus-time curve (AUC) of single 750 mg ciprofloxacin dose were each reduced by 85% relative to ciprofloxacin alone (control) when administered 5–10 min after Maalox ingestion [9]. Nix et al. investigated the effect of sucralfate pretreatment at time intervals of 6 and 2 h prior to a single ciprofloxacin dose with reductions in *C*_max_ and AUC of 30% relative to ciprofloxacin alone [9]. Coadministration of ciprofloxacin and sucralfate was subsequently studied by Garrelts et al., finding reductions in *C*_max_ and AUC of 90% and 87%, respectively, versus control [10]. Sahai et al. probed the effects of magnesium and aluminum found in didanosine on ciprofloxacin PK highlighting significant reductions in both mean *C*_max_ (94%) and AUC (98%) seen versus control [11]. Aluminum has emerged as particularly important among product component salts as aluminum has been observed to modulate quinolone PK to a higher degree than other metals. For example, Sahai et al. investigated the effects of calcium carbonate from supplements on ciprofloxacin bioavailability [12]. While both *C*_max_ and AUC were reduced by about 40%, the comparatively smaller influence on bioavailability is apparent. A number of studies disclose the effects of magnesium, aluminum, and calcium on the PK of other fluoroquinolones, including levofloxacin [13,14], enoxacin [15], lomefloxacin [16], pefloxacin [17], rufloxacin [18], norfloxacin [19,20,21,22], ofloxacin [20,23], and fleroxacin [24].

Sources of these multivalent metals are diverse and quite abundant and can originate from diet or from drug products. The case of significant reduction in drug systemic concentration in going from the fasted to fed state represents a negative food effect [25]. Multivalent metals are found in antacids containing calcium, magnesium, and aluminum ions, in addition to vitamin supplements containing manganese, iron, copper, zinc, and cobalt ions. A typical 30 mL dose of liquid suspension dose of Maalox contains 1.8 g of magnesium hydroxide and 3.6 g of aluminum hydroxide. Similarly, the antacid tablet Kolantyl displays a high concentration of active magnesium and aluminum salts, containing a combination of magnesium trisilicate, magnesium hydroxide, and aluminum hydroxide. Sucralfate, a medication prescribed to treat ulcers, gastroesophageal reflux disease (GERD), and stomach inflammation, contains a ratio of 16 aluminum cations per molecule (~200 mg aluminum per gram sucralfate) in the form of free and sulfur-bound aluminum hydroxide. Buffering agents within product formulations may also contain multivalent metals that contribute to reductions in bioavailability. Didanosine, an antiretroviral therapy for HIV/AIDS, is often formulated alongside dihydroxy aluminum sodium carbonate and magnesium hydroxide (in addition to sodium citrate) to lessen acid hydrolysis within the stomach.

Several clinical failure case studies have been reported due to FQ–multivalent cation interactions and the resulting undesired modulations of PK [26,27,28,29]. These interactions have also been implicated as contributors to increasing FQ-resistant bacterial strains as reduced systemic antibiotic concentrations approach the lower end of the mutant selection window [30,31,32]. Clinical management strategies aimed at reducing or avoiding these interactions during FQ regimens include (1) ensuring the FQ dose and ingested multivalent cations are administered separately and sufficiently far apart in time, (2) halt the patient from ingesting multivalent cation-containing compounds altogether, (3) minimize the frequency of the patient ingesting multivalent cation-containing compounds, and (4) switching from oral to intravenous administration. Despite these guidelines, patient adherence to spaced ingestion of FQ medication and sources of metal cations can be low and outside a practitioner’s control.

Computational approaches can provide detailed structural and mechanistic insight into quinolones and their metal complexes. Previous molecular modeling studies tend to focus on investigating the electronic properties and protonation states of the quinolone structure alone. Density functional theory (DFT) calculations were applied by Vitorino et al. in a study comparing neutral and zwitterionic norfloxacin protonation state energies in water [33]. Similarly, a joint experimental and computational study by Pavez et al. probed the protonation states of nalidixic acid combining fluorescence measurements and DFT calculations to locate energetically stable conformers and ionization forms [34]. Time-dependent DFT calculations and computed spectra by Musa et al. studied the protonation state, photochemistry, and degradation pathways of norfloxacin [35]. Computational investigations that do probe interactions with multivalent metals primarily study alkali, alkali earth, and first-row transition metal complexes. An early example from Aristilde et al. discloses molecular dynamics (MD) simulations of ciprofloxacin in aqueous solvent and investigates the stability of complexes formed between ciprofloxacin and sodium, potassium, calcium, magnesium, and iron(II) [36]. Recent examples include DFT computations by Bridle et al. modeling pH-dependent complexes of ciprofloxacin and magnesium in aqueous solution [37], and an experimental and computational study of ciprofloxacin derivatives complexed with copper(II) [38].

Variable patient compliance with FQ spacing regimens coupled with the need for new structures for applications to FQ-resistant bacteria [39,40,41] points to the utility for continued studies of chelation interactions between FQs and commonly encountered multivalent cations found in diet and medication. Reports of aluminum salts and their effect on bioavailability indicate a higher magnitude of effect compared to other multivalent metals, but thus far computational studies of FQ interactions with aluminum ion remain more limited. Furthermore, decreases in bioavailability due to metal chelation are predominantly attributed to reduced absorption rate [9,42], but direct analysis of absorption rate constants is rarely presented in the literature.

In this report, examples of FQ interactions with aluminum, calcium, and mixed aluminum/magnesium metals are presented and analyzed within the context of PK parameters and calculated oral absorption rate constants extracted from tabulated literature data and plasma concentration–time curves. Two main objectives guided this study: (1) investigate the relationship between FQ absorption rate and alkaline earth and aluminum cation sources, and (2) develop a computational parameter for assessing the extent of bioavailability reduction from FQ structure. Computed binding energies were hypothesized as a predictor for changes in absorption rate and bioavailability due to metal hydrate chelation. We undertook DFT computations of the energies of binding of structurally diverse FQs to aqueous magnesium, calcium, and aluminum, and present relationships between computed chelation energies, molecular descriptors, and relative changes in bioavailability.

## 2. Methods

### 2.1. Oral Absorption Rate Constant Calculations

The oral absorption rate constant, *k*_a_, describes the rate at which a drug moves from the gastrointestinal tract to the plasma and systemic circulation. Calculation of *k*_a_ is achieved by approximating the elimination phase of a one-compartment model and the time to maximum plasma concentration (*T*_max_). Assuming a first-order absorption process, *T*_max_ is related to *k*_a_ and the elimination rate constant, *k*_e_, by Equation (1) [43]:

(1) $$T_{\max}=\frac{\ln\left(\frac{k_{a}}{k_{e}}\right)}{k_{a}-k_{e}}$$

The elimination rate constant can be calculated from the concentration-versus-time curve, where the post-absorption phase of the log scale plot has slope 2*k*_e_/2.303. In the case of a one-compartment model, *k*_e_ may be derived from the terminal slope and used for calculation of the half-life period (t_1/2_). For all compounds, the *k*_e_ was ultimately calculated from *t*_1/2_ values using Equation (2):

(2) $$t_{1/2}=\frac{\ln\left(2\right)\times V_{d}}{CL}=\frac{\ln\left(2\right)}{k_{e}}=\frac{0.693}{k_{e}}$$

where *V*_d_ is the drug’s volume of distribution and *CL* is clearance. If *T*_max_ and *t*_1/2_ were provided as tabulated pharmacokinetic parameters within a literature source, these values were used as given for the *k*_a_ calculation, first using *t*_1/2_ and Equation (2) giving *k*_e_, then solving for *k*_a_ given *T*_max_ and Equation (1). While *T*_max_ was consistently available in primary literature sources, *t*_1/2_ was not always available. In such cases, the pertinent concentration-versus-time curve was digitized and *t*_1/2_ and *k*_a_ were extracted using the slope of the post-absorption phase in the procedure described above, followed by use of Equation (1) yielding *k*_a_. The complete set of data tabulating dose, administration timing, *C*_max_, *T*_max_, *t*_1/2_, AUC, *k*_e_, and *k*_a_ may be found in the Table S1 (Supplementary Materials).

### 2.2. Physicochemical Descriptor Calculations

SMILES strings for all FQ compounds were used to calculate physicochemical property descriptors. SMILES strings ware taken from the PubChem public database [44]. PubChem compound identification numbers and SMILES strings can be found in the Supplementary Materials (Table S2). Topological polar surface area (TPSA) was calculated using the surface contribution-based method of Ertl et al. [45] and assuming nitrogen, oxygen, sulfur, and phosphorus atoms as polar. The calculated logarithm of the octanol/water partition coefficient (log*P*) was predicted using the Wildman–Crippen model [46]. Tabulated physicochemical properties can be found in Table 1. All descriptors were calculated as implemented in the Mordred cheminformatics Python package [47,48].

### 2.3. Molecular Modeling

#### 2.3.1. Metal Chelate Construction

Amphoteric FQs can exist in several protonation states in aqueous solvent [49,50]. At neutral pH, both the carboxylic acid and piperazine nitrogen atom are predominantly ionized, forming a zwitterionic species [51]. To reflect the acidic environments of the stomach and gastrointestinal tract (GIT), the cationic species was used throughout all molecular modeling computations. FQ structures were modeled under acidic conditions assuming unionized 3-carboxyl group and protonated terminal *N*-piperazinyl group in the R7 position. It was assumed that gastric pH remains acidic even after antacid administration [52]. Magnesium, calcium, and aluminum ions were constructed as the octahedral hexahydrate species. Bidentate FQ chelation (3-carboxyl and 4-keto oxygen atoms bound to the metal center) was considered for all computed complexes as observed experimentally from spectroscopy and crystal structures [53,54]. FQ–metal complexes were built as the 1:1 stoichiometry chelates. Under higher metal concentrations or acidic conditions, a 1:1 ratio of FQ to metal is the most observed species [55]. Quinoline–metal chelates can exhibit higher ratios of 2:1 and 3:1 dependent on, among other factors, pH, quinolone structure, and the specific multivalent metal [53,56,57,58]. The energetic favorability of 1:1 chelate formation under the acidic conditions was taken as a quantitative metric for the propensity of FQ–metal interactions that affect bioavailability.

#### 2.3.2. Conformational Search

Three-dimensional coordinates of ciprofloxacin were taken from the PubChem public database [59] and imported into the Avogadro molecular modeling interface [60]. The cationic *N*-protonated species was then constructed and submitted to a conformational search using the MMFF94 force field [61] to locate all pertinent low energy conformers. Starting ciprofloxacin chelate complexes between magnesium, calcium, and aluminum were built and an additional conformational search was performed on the complexes to exhaustively probe the conformational space of the bound water molecules and torsions of the R1 and R7 rings (Figure 1). Recent DFT computations of ciprofloxacin and levofloxacin identified the importance of piperazine ring conformations and potential intramolecular interactions in determining the stability of ground state FQ structures [62].

#### 2.3.3. Quantum Mechanical Computations

FQ and FQ–metal chelate structures from the conformational search were then submitted to geometry optimization using the PBE method [63], 6–31G(d) basis set [64,65], and included the D3 dispersion correction with Becke–Johnson dampening [66,67]. This level of theory is denoted as PBE-D3BJ/6–31G(d). Ground state minima were confirmed at the same level of theory with vibrational frequency calculations to ensure zero imaginary frequencies were present in the optimized structures for magnesium, calcium, and aluminum–ciprofloxacin chelates. Frequencies were computed at a temperature of 310 K, matching approximate conditions within the stomach and GIT. Further single-point energies for all optimized structures were computed using PBE-D3BJ/6–31+G(d,p). Aqueous solvation was modeled implicitly using the polarizable continuum model (PCM) [68], denoted as PBE-D3BJ/6–31+G(d,p)/PCM=water. PCM-corrected single-point energies were used to compute the predicted energy of binding (Δ*E*_bind_) while the sum of the Gibbs free energy thermal correction and the PCM single-point was used to predict the Gibbs free energy of binding (Δ*G*_bind_). All quantum mechanical computations were completed using the Psi4 ab initio quantum chemical computational package [69]. All reported energies are in units of kcal mol^−1^ (1 hartree = 627.5095 kcal mol^−1^), and all labeled distances in structural images are Ångstroms (Å) [70]. Computed energies for all compound species are tabulated in Table S3 (Supplementary Materials).

## 3. Results and Discussion

Measured human in vivo pharmacokinetic parameters comparing control administration (FQ alone) and coadministration with products containing aluminum, magnesium, and calcium cations were extracted from the literature for thirteen FQ compounds. The full set of parameters tabulated and calculated were *C*_max_, *T*_max_, AUC, *t*_1/2_, *k*_e_, and *k*_a_, in addition to dose amounts and precise administration timing (concurrent versus FQ given 1 to 10 minutes after multivalent metal dose). Table 2 summarizes relative percent change in *C*_max_, AUC, and calculated *k*_a_ for each FQ, the co-administered multivalent metal, and associated metal source. The bioavailability of each FQ was reduced in all combinations of multivalent metal and metal sources compared to FQ administered alone. A similar trend was observed for *C*_max_, with the lone exception of rufloxacin, which showed a small increase in *C*_max_ upon administration of Maalox antacid. The small magnitudes of relative change in both *C*_max_ (6.1%) and AUC (–15.2%) for rufloxacin indicate overall little sensitivity to bioavailability changes because of aluminum/magnesium interactions [18]. The most common source of aluminum was aluminum hydroxide originating predominantly from the crossover study of Shiba et al., which tested the effects of aluminum hydroxide on FQ bioavailability [71].

Changes in bioavailability from mixed metal (aluminum/magnesium) antacid Maalox and sucralfate allow comparison to the effects of pure aluminum salts. In general, aluminum hydroxide, Maalox, and sucralfate all exhibited similar effects on PK and changes in bioavailability for the studies reported in this manuscript. Reductions in ciprofloxacin AUC were within experimental variability for Maalox (−84.9%) and aluminum hydroxide metal sources (−84.6% and −87.5%). A greater difference in AUC reduction is observed when comparing aluminum hydroxide versus Maalox co administration for enoxacin (−84.2% versus −73.2%). Norfloxacin AUC measurements were not reported in the original literature source for the dosing regimen of 30 mL Maalox 5 minutes prior to FQ administration [21]. In this case, *C*_max_ values are compared and indicate similar reductions between aluminum hydroxide versus Maalox coadministration (−93.3% versus −95.1%). Aluminum hydroxide and sucralfate comparisons indicate more variability. While similar reductions in AUC are seen for ciprofloxacin (−84.6% and −87.5% versus −87.5%), differences emerge between fleroxacin (−17.2% versus −24.0%) and norfloxacin (−93.3% versus −92.2%), and most significantly, ofloxacin (−47.9% versus −61.0%).

Relative changes in calculated absorption rates constants were much more variable than AUC and *C*_max_, both in magnitude and direction of change upon coadministration of multivalent metals. As a result, the previously hypothesized correspondence between reductions in AUC and reduced *k*_a_ values was not consistently observed. For example, as seen in Table 2, ciprofloxacin co-administered with aluminum hydroxide yielded relative decreases in *k*_a_ values of 14.3% and 60.6% yet observed decreases in AUC from Maalox and didanosine interactions were associated with increases in absorption rate constants of 157.9% and 49.2%, respectively. As a metal source, aluminum hydroxide salt solutions did yield reductions in *k_a_* in the majority of FQ pharmacokinetic studies investigated apart from sparfloxacin, which is associated with a 47.1% increase in *k*_a_ upon metal coadministration. Sucralfate as an aluminum source also showed consistent reductions in *k*_a_ upon concurrent FQ administration with diminished absorption rates of 40.3% (fleroxacin), 23.8% (norfloxacin), 46.2% (ofloxacin), and 80.7% (moxifloxacin).

**Figure 2:**
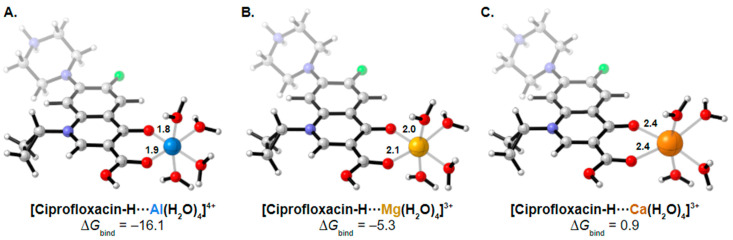
Computed structures and predicted free energies of binding (∆*G*_bind_) between *N*-protonated cationic ciprofloxacin and (**A**) aluminum hydrate, (**B**) magnesium hydrate, and (**C**) calcium hydrate. Structural images generated using CYLView [70].

Analyzing effects on bioavailability across metals highlights calcium ion’s smaller influence compared to aluminum and mixed aluminum/magnesium sources. Ciprofloxacin coadministered with calcium carbonate antacid Titralac leads to a 41% reduction in AUC compared to ciprofloxacin alone (Table 2), but this observed reduction in AUC is close to half that of aluminum and magnesium (85% to 98% reduction). A similar difference in AUC relative change is seen for norfloxacin (>90% versus 63% reduction), and most significantly with moxifloxacin, where calcium (Calcium-Sandoz) has little effect entirely on bioavailability (−59.9% versus −2.3%). Figure 2 shows the computed octahedral bidentate chelate structures of cationic *N*-protonated ciprofloxacin 3-keto and 4-carboxyl groups bound to aluminum, magnesium, and calcium tetrahydrate. The trend in predicted free energy of binding (Δ*G*_bind_) in order of decreasing ciprofloxacin–metal chelate stability is aluminum (−16.1 kcal mol^−1^) > magnesium (−5.3 kcal mol^−1^) > calcium (0.9 kcal mol^−1^). The energetic trend is reflected in the 4-keto and 3-carboxyl oxygen–metal bond lengths as an indication of bond strength. The strongest bonds are formed between aluminum (C3=O∙∙∙Al = 1.9 Å, C4=O∙∙∙Al = 1.8 Å) and the weakest between calcium (C3=O∙∙∙Ca = 2.3 Å, C4=O∙∙∙Ca = 2.4 Å) as function of ion charge density [56,76]. Aluminum ion exhibits a higher positive charge distributed over a smaller volume, while calcium ion is the least charge dense, and the FQ oxygen–metal bonds lengthen to avoid disfavored bond angle distortion with the larger calcium ion.

**Figure 3:**
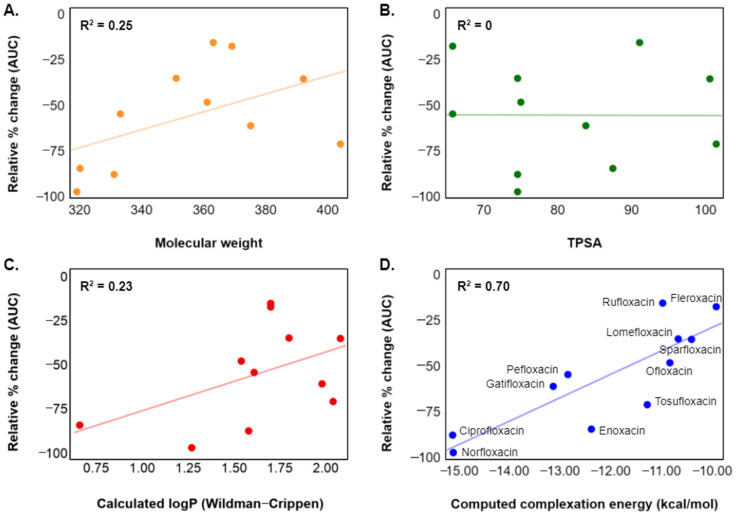
Plots of relative percent change in bioavailability (AUC) versus (**A**) molecular weight, (**B**) topological polar surface area (TPSA) [45], (**C**) predicted log*P* from chemical structure (Wildman–Crippen model) [46], and (**D**) computed complexation energy (Δ*E*_bind_) to aluminum hexahydrate. Changes in AUC on the *y*-axis are from FQ coadministration with aluminum hydroxide [71] or Maalox.

Relationships between FQ physicochemical properties and changes in bioavailability were investigated to provide insight into the structural properties that drive modulations in PK behavior upon metal coadministration (Figure 3A–C). The plotted changes in AUC are primarily taken from concurrent administration of aluminum hydroxide solutions from Shiba et al. [71], with Maalox administration used for compounds without aluminum hydroxide data. For AUC reductions derived from aluminum hydroxide shown in Figure 3, all FQ doses were 200 mg (except tosufloxacin, which was 150 mg) and all aluminum hydroxide doses were 1 g aluminum hydroxide gel (99%). Three widely applied parameters for developing absorption and bioavailability quantitative structure–property relationships (QSPRs) are molecular weight (MW), TPSA, and the logarithm of a compound’s octanol/water partition coefficient (log*P*) [77,78]. Plotting relative change in AUC versus each of these descriptors yielded relationships ranging from no correlation (TPSA, R^2^ = 0) to moderate correlation (calculated log*P* and MW, R^2^ = 0.23 and 0.25, respectively). Slight correspondences are observed between both decreasing molecular weight and log*P* and reduction in AUC. A possible deficiency in these predicted descriptors is a lack of sensitivity to the change in molecular properties upon chelation (e.g., for calculated log*P*, Δlog*P* = log*P*(chelate) ‒ log*P*(FQ) = constant across all structures). Even so, calculation of these descriptors from the parent structure may still act as proxies to more complex three-dimensional, electronic, and mechanistic effects.

FQ chelation to metals represents an equilibrium process dependent on the energetic stability of both the parent FQ and the resulting metal complex. In this study, a strong relationship was observed between percent reduction in AUC and the computed energies of FQ–metal complex formation (Figure 3D, R^2^ = 0.70). These results demonstrate an in silico connection between FQ–metal equilibria and decreases in overall bioavailability. Early experimental work investigating FQ absorption in the presence of the metal ions proposed that the reduction of FQ absorption is due to formation of insoluble and unabsorbable chelates in the GIT [79]. Further studies observed that the solubility of FQs actually increases in the presence of calcium, magnesium, aluminum, and iron(III) [56]. These findings suggest that the reduction of the gastric absorption of lomefloxacin at co-administration with these metal ions are not caused by the insoluble precipitation, but by a decrease in permeability. FQs which experience dramatic decreases in bioavailability due to metal complex formation may be considered class 3 products (high solubility, low permeability) according to the Biopharmaceutics Classification System (BCS) [80,81]. Aluminum complexation can act as a solubilizer but converts the active ingredient into a form that is less permeable through the intestinal lumen [56,82]. This phenomenon is a significant component of the complete mechanistic picture of bioavailability reduction due to metal chelation [58,83,84].

Novel and more structurally diverse FQs will continue to be developed for application to challenging bacterial infections with increasing antibiotic resistance. Predicting potential drug–drug interactions and quantifying the extent is a significant hurdle as new FQs enter the drug development phase [85]. While spacing the administration of the multivalent metals and FQs serves as a general guide in clinical practice, the effect of chelation on FQ pharmacokinetics remains highly variable (e.g., rufloxacin, which only shows a 15% decrease in AUC upon aluminum coadministration, Table 2) and patient compliance cannot be guaranteed. We have highlighted FQ–aluminum binding energy, a simple metric for assessing the magnitude of bioavailability reduced from FQ–aluminum chelation. The computational chemistry workflow outlined herein used free and open-source tools at all steps, and the methodology could easily be applied early in the drug development pipeline. As the model presented has only been developed for aluminum, further research will expand this methodology to other metals known to chelate FQs and disrupt pharmacokinetics such as iron and copper. These QSPR models will then be incorporated into our integrated mechanistic modeling and artificial intelligence platform, BIOiSIM, for predicting complete drug disposition [86,87].

## 4. Conclusions

A multitude of literature sources disclose reductions in bioavailability of fluoroquinolone (FQ) antibiotics due to interactions with the multivalent metals magnesium, calcium, and aluminum commonly found in antacids and drug formulations. Of these metals, aluminum is shown to affect pharmacokinetic (PK) parameters the highest. Literature sources for PK studies of FQ–metal interactions and effects on the PK parameters *C*_max_, AUC, and *t*_1/2_ were leveraged to calculate the oral absorption rate constants for thirteen FQs alone in the presence of magnesium, calcium, and aluminum. Rates of absorption were found to generally decrease with concomitant metal administration, with exceptions dependent on the identity of the drug product. Quantum mechanical computations predicted the Gibbs free energy of binding (∆*G*_bind_) of ciprofloxacin to magnesium, calcium, and aluminum ions under aqueous acidic conditions, showing good agreement with trends in relative AUC reduction. Descriptors commonly applied in absorption prediction models (molecular weight, polar surface area, and log*P*) were identified as poor-to-moderate predictors of AUC reduction of FQs due in the presence of aluminum. The predicted energy of binding (∆*E*_bind_) corresponded well with reductions in bioavailability for a structurally-diverse set of FQs. A straightforward computational metric has been developed to evaluate metal chelation interaction propensity and the magnitude of the resulting diminished bioavailability that may be applied to novel FQs in clinical development.

## Figures and Tables

**Table 1:** Physicochemical properties of the fluoroquinolones studied in this manuscript.

Generation	Fluoroquinolone	Structure	MW	TPSA	Calculated log*P*
Second	Ciprofloxacin	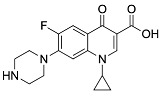	331.13	74.57	1.58
Second	Enoxacin	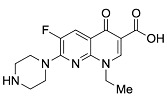	320.13	87.46	0.66
Second	Fleroxacin	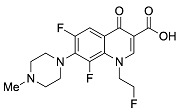	369.13	65.78	1.70
Second	Lomefloxacin	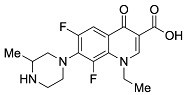	351.14	74.57	1.80
Second	Norfloxacin	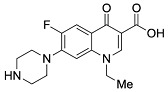	319.13	74.57	1.27
Second	Ofloxacin	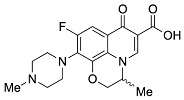	361.14	75.01	1.54
Second	Pefloxacin	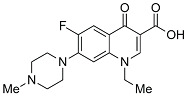	333.15	65.78	1.61
Second	Rufloxacin	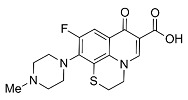	363.11	91.08	1.70
Third	Levofloxacin	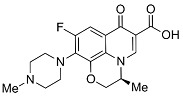	361.14	75.01	1.54
Third	Sparfloxacin	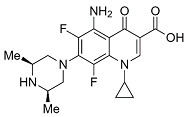	392.17	100.59	2.08
Third	Tosufloxacin	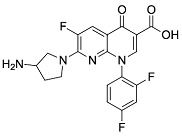	404.11	101.45	2.04
Fourth	Gatifloxacin	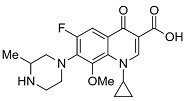	375.16	83.8	1.98
Fourth	Moxifloxacin	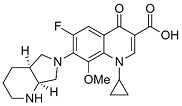	401.18	83.8	2.37

MW: molecular weight (g/mol); TPSA: topological polar surface area in square Ångstroms (Å^2^) with O, N, S, P atoms considered polar; log*P*: logarithm of the partition coefficient between octanol and water; See Section 2.2 for additional details on physicochemical property calculations.

**Table 2:** Relative change of fluoroquinolone pharmacokinetic parameters upon coadministration with antacids and drug products containing multivalent metals.

			Relative Change (%)			
Fluoroquinolone	Multivalent Metal	Metal Source	*C*_max_	AUC	Calculated *k*_a_	Reference
Ciprofloxacin	Aluminum	Aluminum hydroxide	−81.1	−84.6	−14.3	[72]
	Aluminum	Aluminum hydroxide	−84.6	−87.5	−60.6	[71]
	Aluminum	Sucralfate	−90.0	−87.5	N/A	[10]
	Aluminum/Magnesium	Maalox	−80.1	−84.9	157.9	[9]
	Aluminum/Magnesium	Didanosine	−92.6	−98.3	49.2	[11]
	Calcium	Titralac	−37.9	−41.1	5.9	[12]
Enoxacin	Aluminum	Aluminum hydroxide	−78.3	−84.2	N/A	[71]
	Aluminum/Magnesium	Maalox	−70.0	−73.2	−52.1	[15]
Fleroxacin	Aluminum	Aluminum hydroxide	−25.0	−17.2	−47.6	[71]
	Aluminum	Sucralfate	−26.4	−24.0	−40.3	[24]
Lomefloxacin	Aluminum	Aluminum hydroxide	−54.5	−34.8	−55.1	[71]
	Aluminum	Kolantyl	−46.1	−40.8	25.6	[16]
Norfloxacin	Aluminum	Aluminum hydroxide	−93.3	−97.0	N/A	[71]
	Aluminum	Sucralfate	−92.2	−91.3	−23.8	[20]
	Aluminum/Magnesium	Maalox	−95.1	N/A	−6.1	[21]
	Calcium	Titralac	−65.9	−62.6	−80.2	[21]
Ofloxacin	Aluminum	Aluminum hydroxide	−59.4	−47.9	−65.0	[71]
	Aluminum	Sucralfate	−69.5	−61.0	−46.2	[20]
Pefloxacin	Aluminum/Magnesium	Maalox	−60.8	−54.3	−57.5	[17]
Rufloxacin	Aluminum/Magnesium	Maalox	6.1	−15.2	−34.0	[18]
Levofloxacin	Aluminum	Aluminum hydroxide	−66.7	−45.2	−55.5	[71]
Sparfloxacin	Aluminum	Aluminum hydroxide	−22.2	−35.1	47.1	[71]
Tosufloxacin	Aluminum	Aluminum hydroxide	−66.7	−70.8	−67.4	[71]
Gatifloxacin	Aluminum/Magnesium	Maalox	−68.4	−60.8	−13.5	[73]
Moxifloxacin	Aluminum	Sucralfate	−79.5	−59.9	−80.7	[74]
	Calcium	Calcium-Sandoz	−15.5	−2.4	−74.1	[75]

N/A: not available; *C*_max_: maximum plasma concentration (µg∙mL^−1^); AUC: area-under-the-curve of the concentration plasma profile (µg∙h∙mL^−1^); Calculated *k*_a_: calculated absorption rate constant (h^−1^) using Equations (1) and (2) and the methods outlined in Section 2.1 of this manuscript.

## Data Availability

Not applicable.

## Supplementary Materials

The following are available online at https://www.mdpi.com/article/10.3390/pharmaceutics13050594/s1, Table S1: Literature and calculated (where applicable) pharmacokinetic parameters including dose, administration timing, *C*_max_, *T*_max_, *t*_1/2_, AUC, *k*_e_, and *k*_a_, Table S2: PubChem compound identification number (CID) and SMILES, Table S3: Computed compound species and metal chelate complex energies.
