# Supplementary material for: Effects of Magnesium, Calcium, and Aluminum Chelation on Fluoroquinolone Absorption Rate and Bioavailability: A Computational Study
Source: Pharmaceutics. 2021 Apr 21;13(5):594. doi: 10.3390/pharmaceutics13050594 (PMC8143323; doi:10.3390/pharmaceutics13050594)
Supplement: Supplementary file 1 [file pharmaceutics-13-00594-s001.zip › pharmaceutics-1185805-supplementary.pdf]

pharmaceutics

MDPI

Supplementary Materials: Effects of Magnesium, Calcium, and Aluminum Chelation on Fluoroquinolone Absorption Rate and Bioavailability: A Computational Study

Daniel M. Walden, Maksim Khotimchenko, Hypatia Hou, Kaushik Chakravarty and Jyotika Varshney

Table 1. Fluoroquinolone pharmacokinetic parameters upon coadministration with antacids and drug products containing multivalent metals.

Fluoroquinolone	Dose Regimen	C_{max} (ug/mL)	T_{max} (h)	$t_{1/2}$ (h)	AUC (μg h mL^{-1})	$t_{1/2}$ calc. (h)	k_e calc. (h^{-1})	k_a calc. (h^{-1})	Reference
Ciprofloxacin	750 mg	3.18	1.24	3.9	13.5	3.9	0.18	2.21	[9]
	750 mg 5 mins after 3 x 600 mg aluminum hydroxide	0.6	1.61	6.5	2.08	6.5	0.11	1.89	
	200 mg ciprofloxacin	1.30	1.30	2.40	6.40	2.40	0.29	1.61	[10]
	200 mg concurrent with 1 g aluminum hydroxide	0.20	2.10	2.00	0.80	2.00	0.35	0.63	
	500 mg	2.0	1.4	-	8.8	4.42	0.16	1.96	[11]
	500 mg concurrent with 1 g sucralfate	0.2	1.3	-	1.1	N/A	N/A	N/A	
	750 mg	3.42	1.25	3.83	16.07	3.83	0.18	2.17	[12]
	750 mg 5-10 mins after 30 mL Maalox	0.68	0.66	4.48	2.42	4.48	0.15	5.59	
	750 mg	3.38	1.56	4.29	15.5	N/A	0.16	1.65	[15]
	750 mg concurrent with didanosine	0.25	0.75	-	0.26	1.13	0.62	2.47	
	500 mg	2.9	-	-	12.4	3.53	0.20	2.90	[16]
	500 mg concurrent with 500 mg calcium	1.8	-	-	7.3	4.06	0.17	3.08	
Enoxacin	200 mg	2.30	0.80	3.90	11.40	N/A	0.18	4.10	[10]
	200 mg concurrent with 1 g aluminum hydroxide	0.50	1.60	-	1.80	N/A	N/A	N/A	
	400 mg	3.17	1.00	3.78	14.50	N/A	0.18	2.97	[17]
	400 mg concurrent with 30 mL Maalox	0.95	1.50	2.86	3.89	N/A	0.24	1.42	
Fleroxacin	200 mg	2.40	0.80	10.90	32.60	10.90	0.06	5.68	[10]
	200 mg concurrent with 1 g aluminum hydroxide	1.80	1.30	10.20	27.00	N/A	0.07	2.98	
	400 mg	5.30	1.70	-	70.30	10.15	0.07	2.08	[18]
	400 mg concurrent with 1 g sucralfate	3.90	2.50	-	53.40	10.54	0.07	1.24	
Lomefloxacin	200 mg	2.20	1.10	3.90	13.50	N/A	0.18	2.63	[10]
	200 mg concurrent with 1 g aluminum hydroxide	1.00	2.00	4.60	8.80	N/A	0.15	1.18	
	200 mg	1.91	1.60	6.83	12.64	N/A	0.10	1.95	[20]
	200 mg concurrent with 2 g Kolantyl	1.03	1.40	7.67	7.48	N/A	0.09	2.45	
Norfloxacin	200 mg	1.50	1.20	2.90	6.70	N/A	0.24	2.02	[10]
	200 mg concurrent with 1 g aluminum hydroxide	0.10	-	-	0.20	N/A	N/A	N/A	
	400 mg	1.29	1.60	-	7.39	5.78	0.12	1.82	[21]
	400 mg concurrent with 1 g sucralfate	0.10	1.80	-	0.64	4.63	0.15	1.39	

	400 mg	1.64	1.25	-	6.69	3.91	0.18	2.19	[24]
	400 mg 5 mins after 30 mL Maalox	0.08	1.56	-	-	7.14	0.10	2.05	
	400 mg 5 mins after 30 mL calcium carbonate	0.56	2.77	-	2.50	2.34	0.30	0.43	
Ofloxacin	200 mg	3.20	1.10	5.10	23.80	N/A	0.14	2.93	[10]
	200 mg concurrent with 1 g aluminum hydroxide	1.30	2.40	6.00	12.40	N/A	0.12	1.03	
	400 mg	3.05	1.60	-	29.50	4.63	0.15	1.65	[21]
	400 mg concurrent with 1 g sucralfate	0.93	2.90	-	11.50	7.95	0.09	0.89	
Pefloxacin	400 mg	5.10	1.05	10.60	56.50	N/A	0.07	3.98	[71]
	400 mg concurrent with 30 mL Maalox	2.00	1.95	9.60	25.80	N/A	0.07	1.69	
Rufloxacin	400 mg	3.74	2.50	39.00	178.00	N/A	0.02	1.88	[72]
	400 mg 5 mins after 30 mL Maalox	3.97	3.50	40.80	151.00	N/A	0.02	1.24	
Levofloxacin	100 mg	1.80	0.80	6.40	9.30	N/A	0.11	4.86	[10]
	100 mg concurrent with 1 g aluminum hydroxide	0.60	1.50	7.10	5.10	N/A	0.10	2.16	
Sparfloxacin	200 mg	0.90	5.30	14.70	21.10	N/A	0.05	0.49	
	200 mg concurrent with 1 g aluminum hydroxide	0.70	4.00	14.00	13.70	N/A	0.05	0.72	
Tosufloxacin	150 mg	0.30	1.30	3.30	2.40	N/A	0.21	1.91	
	150 mg concurrent with 1 g aluminum hydroxide	0.10	3.50	6.90	0.70	N/A	0.10	0.62	
Gatifloxacin	400 mg	3.80	1.40	8.60	33.50	N/A	0.08	2.55	[73]
	400 mg concurrent with 600 mg magnesium hydroxide/900 mg of aluminum hydroxide	1.20	1.60	9.50	11.90	N/A	0.07	2.20	
Moxifloxacin	400 mg	2.83	1.00	12.90	32.20	N/A	0.05	4.48	[74]
	400 mg 5 mins after 1 g sucralfate	0.58	3.50	13.90	12.90	N/A	0.05	0.87	
	400 mg	2.71	0.88	13.20	33.00	N/A	0.05	5.30	[75]
	400 mg concurrent with 500 mg Calcium-Sandoz	2.29	2.50	13.70	32.20	N/A	0.05	1.37	

N/A: not available; C_{max}: maximum plasma concentration; T_{max}: time to maximum plasma concentration; $t_{1/2}$: half-life; AUC: area-under-the-curve of the concentration plasma profile; $t_{1/2}$ calc.: calculated half-life; k_e calc.: elimination rate constant; k_a calc.: absorption rate constant. All calculated values generated using the methods outlined in Section 2.1 of the manuscript

Table S2. Fluoroquinolones PubChem compound identification numbers and SMILES strings.

Fluoroquinolone	PubChem CID	SMILES
Ciprofloxacin	2764	C1CC1N2C=C(C(=O)C3=CC(=C(C=C32)N4CCNCC4)F)C(=O)O
Enoxacin	3229	CCN1C=C(C(=O)C2=CC(=C(N=C21)N3CCNCC3)F)C(=O)O
Fleroxacin	3357	CN1CCN(CC1)C2=C(C=C3C(=C2F)N(C=C(C3=O)C(=O)O)CCF)F
Lomefloxacin	3948	CCN1C=C(C(=O)C2=CC(=C(C(=C21)F)N3CCNC(C3)C)F)C(=O)O
Norfloxacin	4539	CCN1C=C(C(=O)C2=CC(=C(C=C21)N3CCNCC3)F)C(=O)O
Ofloxacin	4583	CC1COC2=C3N1C=C(C(=O)C3=CC(=C2N4CCN(CC4)C)F)C(=O)O
Pefloxacin	51081	CCN1C=C(C(=O)C2=CC(=C(C=C21)N3CCN(CC3)C)F)C(=O)O
Rufloxacin	58258	CN1CCN(CC1)C2=C(C=C3C4=C2SCCN4C=C(C3=O)C(=O)O)F
Sparfloxacin	60464	C[C@@H]1CN(C[C@@H](N1)C)C2=C(C(=C3C(=C2F)N(C=C(C3=O)C(=O)O)C4CC4)N)F
Tosufloxacin	5517	C1CN(CC1N)C2=C(C=C3C(=O)C(=CN(C3=N2)C4=C(C=C(C=C4)F)F)C(=O)O)F
Gatifloxacin	5379	CC1CN(CCN1)C2=C(C=C3C(=C2OC)N(C=C(C3=O)C(=O)O)C4CC4)F

Table S3. All computed compound species and energies (PBE-D3BJ/6-31+G(d,p)/PCM=water).

Compound	Computed energy (hartrees)
Magnesium hexahydrate	658.0011708
Calcium hexahydrate	−1135.414984
Aluminum hexahydrate	−700.1485534
Water dimer	−152.7241455
Ciprofloxacin (*N*-protonated cation)	−1147.679747
Ciprofloxacin (*N*-protonated cation) magnesium tetrahydrate complex	−1652.963524
Ciprofloxacin (*N*-protonated cation) calcium tetrahydrate complex	−2130.371626
Ciprofloxacin (*N*-protonated cation) aluminum tetrahydrate complex	−1695.128283
Enoxacin (*N*-protonated cation)	−1125.701515
Enoxacin (*N*-protonated cation) aluminum tetrahydrate complex	−1673.145694
Fleroxacin (*N*-protonated cation)	−1347.201465
Fleroxacin (*N*-protonated cation) aluminum tetrahydrate complex	−1894.641722
Lomefloxacin (*N*-protonated cation)	−1248.063014
Lomefloxacin (*N*-protonated cation) aluminum tetrahydrate complex	−1795.504464
Norfloxacin (*N*-protonated cation)	−1109.649136
Norfloxacin (*N*-protonated cation) aluminum tetrahydrate complex	−1657.097653
Ofloxacin (*N*-protonated cation)	−1262.129514
Ofloxacin (*N*-protonated cation) aluminum tetrahydrate complex	−1809.571226
Pefloxacin (*N*-protonated cation)	−1148.913311
Pefloxacin (*N*-protonated cation) aluminum tetrahydrate complex	−1696.358226
Rufloxacin (*N*-protonated cation)	−1545.728828
Rufloxacin (*N*-protonated cation) aluminum tetrahydrate complex	−2093.170767
Sparfloxacin (*N*-protonated cation)	−1380.671223
Sparfloxacin (*N*-protonated cation) aluminum tetrahydrate complex	−1928.112256
Tosufloxacin (*N*-protonated cation)	−1476.273000
Tosufloxacin (*N*-protonated cation) aluminum tetrahydrate complex	−2023.715423
Gatifloxacin (*N*-protonated cation)	−1301.341616
Gatifloxacin (*N*-protonated cation) aluminum tetrahydrate complex	−1848.786992

References

9. Frost, R.W.; Lasseter, K.C.; Noe, A.J.; Shamblen, E.C.; Lettieri, J.T. Effects of aluminum hydroxide and calcium carbonate antacids on the bioavailability of ciprofloxacin. *Antimicrob. Agents Chemother.* **1992**, *36*, 830–832, doi:10.1128/aac.36.4.830.
10. Shiba, K.; Sakamoto, M.; Nakazawa, Y.; Sakai, O. Effects of Antacid on Absorption and Excretion of New Quinolones. *Drugs* **1995**, *49*, 360–361, doi:10.2165/00003495-199500492-00098.
11. Garrelts, J.C.; Godley, P.J.; Peterie, J.D.; Gerlach, E.H.; Yakshe, C.C. Sucralfate significantly reduces ciprofloxacin concentrations in serum. *Antimicrob. Agents Chemother.* **1990**, *34*, 931–933, doi:10.1128/aac.34.5.931.
12. E Nix, D.; A Watson, W.; E Lener, M.; Frost, R.W.; Krol, G.; Goldstein, H.R.; Lettieri, J.T.; Schentag, J.J. Effects of aluminum and magnesium antacids and ranitidine on the absorption of ciprofloxacin. *Clin. Pharmacol. Ther.* **1989**, *46*, 700–705, doi:10.1038/clpt.1989.207.
15. Sahai, J.; Gallicano, K.; Oliveras, L.; Khaliq, S.; Hawley-Foss, N.; Garber, G. Cations in the didanosine tablet reduce ciprofloxacin bioavailability. *Clin. Pharmacol. Ther.* **1993**, *53*, 292–297, doi:10.1038/clpt.1993.24.

16. Sahai, J.; Healy, D.P.; Stotka, J.; E Polk, R. The influence of chronic administration of calcium carbonate on the bioavailability of oral ciprofloxacin. *Br. J. Clin. Pharmacol.* **1993**, *35*, 302–304.
17. Grasela, T.H.; Schentag, J.J.; Sedman, A.J.; Wilton, J.H.; Thomas, D.J.; Schultz, R.W.; E Lebsack, M.; Kinkel, A.W. Inhibition of enoxacin absorption by antacids or ranitidine. *Antimicrob. Agents Chemother.* **1989**, *33*, 615–617, doi:10.1128/aac.33.5.615.
18. Lubowski, T.J.; Nightingale, C.H.; Sweeney, K.; Quintiliani, R. Effect of sucralfate on pharmacokinetics of fleroxacin in healthy volunteers. *Antimicrob. Agents Chemother.* **1992**, *36*, 2758–2760, doi:10.1128/aac.36.12.2758.
20. Shimada, J.; Shiba, K.; Oguma, T.; Miwa, H.; Yoshimura, Y.; Nishikawa, T.; Okabayashi, Y.; Kitagawa, T.; Yamamoto, S. Effect of antacid on absorption of the quinolone lomefloxacin. *Antimicrob. Agents Chemother.* **1992**, *36*, 1219–1224, doi:10.1128/aac.36.6.1219.
21. Lehto, P.; Kivisto, K.T. Effect of sucralfate on absorption of norfloxacin and ofloxacin. *Antimicrob. Agents Chemother.* **1994**, *38*, 248–251, doi:10.1128/aac.38.2.248.
24. Nix, D.; Wilton, J.H.; Ronald, B.; Distlerath, L.; Williams, V.C.; Norman, A. Inhibition of norfloxacin absorption by antacids. *Antimicrob. Agents Chemother.* **1990**, *34*, 432–435, doi:10.1128/aac.34.3.432.
71. Jaehde, U.; Sörgel, F.; Stephan, U.; Schunack, W. Effect of an antacid containing magnesium and aluminum on absorption, metabolism, and mechanism of renal elimination of pefloxacin in humans. *Antimicrob. Agents Chemother.* **1994**, *38*, 1129–1133, doi:10.1128/aac.38.5.1129.
72. Lazzaroni, M.; Imbimbo, B.P.; Bargiggia, S.; Sangaletti, O.; Bo, L.D.; Broccali, G.; Porro, G.B. Effects of magnesium-aluminum hydroxide antacid on absorption of rufloxacin. *Antimicrob. Agents Chemother.* **1993**, *37*, 2212–2216, doi:10.1128/aac.37.10.2212.
73. Lober, S.; Ziege, S.; Rau, M.; Schreiber, G.; Mignot, A.; Koeppe, P.; Lode, H. Pharmacokinetics of Gatifloxacin and Interaction with an Antacid Containing Aluminum and Magnesium. *Antimicrob. Agents Chemother.* **1999**, *43*, 1067–1071, doi:10.1128/aac.43.5.1067.
74. Stass, H.; Schühly, U.; Möller, J.-G.; Delesen, H. Effects of Sucralfate on the Oral Bioavailability of Moxifloxacin, a Novel 8-Methoxyfluoroquinolone, in Healthy Volunteers. *Clin. Pharmacokinet.* **2001**, *40*, 49–55, doi:10.2165/00003088-200140001-00007.
75. Stass, H.; Wandel, C.; Delesen, H.; Möller, J.-G. Effect of Calcium Supplements on the Oral Bioavailability of Moxifloxacin in Healthy Male Volunteers. *Clin. Pharmacokinet.* **2001**, *40*, 27–32, doi:10.2165/00003088-200140001-00004.
